# Testosterone Replacement Therapy in Hypogonadal Men and Myocardial Infarction Risk: Systematic Review & Meta-Analysis

**DOI:** 10.7759/cureus.17475

**Published:** 2021-08-26

**Authors:** Jun Hee Lee, Prutha H Shah, Davuluri Uma, Dhairya J Salvi, Rizwan Rabbani, Pousette Hamid

**Affiliations:** 1 Internal Medicine, California Institute of Behavioral Neurosciences & Psychology, Fairfield, USA; 2 Nephrology, California Institute of Behavioral Neurosciences & Psychology, Fairfield, USA; 3 Neurology, California Institute of Behavioral Neurosciences & Psychology, Fairfield, USA

**Keywords:** testosterone, myocardial infarction, testosterone hormone, hypogonadism, trt, testosterone replacement therapy, mi, hypogonadal men, cardiovascular outcome

## Abstract

Testosterone replacement therapy (TRT) has become increasingly popular over the years and there has been an increasing debate on whether testosterone replacement should be offered to older men due to its association with cardiovascular events. In this study, we evaluated the risk of myocardial infarction (MI) associated with TRT in hypogonadal men through a meta-analysis. We carried out the analysis by following the Preferred Reporting Items for Systematic Review and Meta-Analyses (PRISMA) guidelines and conducted a literature search utilizing the following databases: Google Scholar, PubMed, Science Direct, Cochrane Library trials, and ClinicalTrials.gov. The search strategy resulted in a total of 782 articles, after applying our inclusion and exclusion criteria. Six observational studies and two randomized controlled trials (RCTs) were included for the analysis. A total of 55,806 hypogonadal men with baseline testosterone levels <300ng/mL were included in the analysis. The intervention group received testosterone in various routes including transdermal patches, gels, mouth patches, testosterone injections, and deposits. The incidence of MI was taken to be the primary measure outcomes. The pooled data from eight studies showed MI incidence in 249 out of 11,720 (2.1%) in the TRT group and 1420 out of 33,086 (4.3%) in the control group. The pooled OD showed no statistically significant association of TRT and MI compared to the control group (OR = 0.76, 95% CI 0.36-1.31; p=0.48). The model revealed high heterogeneity with I^2^ =79%. With sensitivity analysis and, excluding two studies out of the eight, the pooled data was able to achieve low heterogeneity with I^2^ = 0%. The newly pooled data from six studies showed MI incidence in 226 out of 10,137 (2.2%) in the TRT group and 969 out of 36,304 (2.7%) in the control group. The pooled OD shows no statistical significance in the association between TRT treatment and MI compared to the control group. (OR =0.87, 95% CI 0.75-1.01; P =0.08). It appears that TRT does not increase the risk of MI as compared to the non-intervention group. Further RCTs with greater population size are needed that could produce more solid results, allowing more definitive conclusions to be made on this topic.

## Introduction and background

Hypogonadism in men is a growing burden for the population around the world. It is estimated that the overall prevalence rate of hypogonadism in men for the general population in the United States (US) is between 3.8-20.4%, Chile 28.1%, Germany 3.4-5%, Finland 19.8%, Malaysia 6-16.1%, Taiwan 12.0%, and Hong Kong 9.5% [1].

Testosterone is the primary sex hormone in males and it is produced mainly by the Leydig cells of the testes in men under the stimulation by luteinizing hormone. In a normal male, approximately 7mg of testosterone is produced daily and the testis produces 95% of circulating testosterone [2]. Homeostasis of testosterone levels is achieved by the hypothalamic-pituitary-gonadotropin axis. Primary hypogonadism is caused by disruption at the level of the testis whilst secondary form is due to disruption at the level of the hypothalamus and pituitary. A decline in testosterone level in older men is associated with impaired mobility, lower muscle strength, and a decrease in muscle mass [3,4]. Testosterone replacement therapy (TRT) has been shown to have benefits on sexual function, mood and depressive symptoms, physical performance, and an increase in lean body mass [5-7]. In the US, approximately 31% of men experience sexual dysfunction [8].

With the combination of the aging population with hypogonadism and the shown benefits of testosterone, TRT has become increasingly popular over the years. In the US alone, annual testosterone spending has increased around four-folds between 2007 and 2016, expenditure had risen from $108 million to $402 million [9]. The use of testosterone is not without side effects. Some of the more established possible adverse effects relating to TRT include polycythemia, fluid retentions, exacerbation of sleep apnea, gynecomastia, and liver toxicity [10-12].

American Urological Association (AUA) suggests that the diagnosis of testosterone deficiency should be achieved by a total testosterone level lower than 300ng/dL measured on two separate occasions in patients who present with symptoms of hypogonadism [13]. Physicians encountering men with androgen deficiency who presents with suggestive symptoms, sexual dysfunction, decreased muscle strength, and poor quality of life may be interested in using testosterone for treatment. However, an unestablished clear relationship between cardiovascular consequences of TRT may be a limiting factor when weighing the possible risks and benefits of the treatment. AUA guideline recommends that cardiovascular disease risk assessment should be done before the start of the testosterone therapy for hypogonadal men. The guideline, however, also states that there is no definitive evidence between the relationship between testosterone therapy and the risk of cardiovascular events and, hence, there is no clear guideline associated with it [13].

It has been shown that testosterone can induce coronary artery relaxation, a direct androgen effect on the vessels independent of conversion to estrogen [14]. Mathur et al. demonstrated long-term testosterone therapy significantly delayed time to exercise-induced myocardial infarction (MI) [15], whilst other studies have shown testosterone therapy and its possible association with increased thrombotic events [16].

The cardiovascular safety of TRT has been debated over the years with mixed results in the literature. In this meta-analysis, we are hoping to answer this question by finding the possible relationship between TRT used in hypogonadal men and the risk of MI. We want to establish evidence-based effects (benefits or risks) associated with testosterone therapy that can aid clinicians in discussing informed discussion about testosterone therapy and the risk of MI with their patients. In doing so, we systematically reviewed the best available evidence about the relationship between testosterone use and the incidences of MI in men.

## Review

Methodology

This meta-analysis was carried out in the accordance with the Preferred Reporting Items for Systematic Reviews and Meta-Analyses (PRISMA) guidelines [17].

Databases

Systemized search of the articles was done in PubMed, Google Scholar, Science direct and Cochrane Library trials ,and ClinicalTrials.gov. Only articles published in the English language were considered. The last search was done on April 17, 2021. The following search terms were used in combination: “Testosterone replacement”, “Testosterone”, “Testosterone therapy” “hypogonadism”, “Myocardial infarction”, “Cardiovascular event”, “MACE”, and “adverse event”. On PubMed, MeSH (Medical Subject Headings) search strategy was used: Myocardial infarction OR Coronary artery disease OR Heart attack AND Testosterone replacement therapy OR Testosterone therapy OR Testosterone AND Hypogonadal men OR hypogonadism AND ("Myocardial Infarction/epidemiology" (Majr) OR "Myocardial Infarction/statistics and numerical data" (Majr) OR "Myocardial Infarction/therapy"(Majr)) OR ("Myocardial Infarction/epidemiology" (Majr) OR "Myocardial Infarction/statistics and numerical data" (Majr) OR "Myocardial Infarction/therapy" (Majr)) OR ("Myocardial Infarction/epidemiology" (Mesh:NoExp) OR "Myocardial Infarction/statistics and numerical data" (Mesh:NoExp) OR "Myocardial Infarction/therapy" (Mesh:NoExp)) AND (("Testosterone/adverse effects" (Majr) OR "Testosterone/deficiency" (Majr) OR "Testosterone/therapeutic use" (Majr) OR "Testosterone/therapy" (Majr)) OR ("Testosterone/adverse effects" (Mesh:NoExp) OR "Testosterone/deficiency" (Mesh:NoExp) OR "Testosterone/therapeutic use" (Mesh:NoExp) OR "Testosterone/therapy" (Mesh:NoExp)) AND ("Hypogonadism/classification" (Majr) OR "Hypogonadism/drug therapy" (Majr) OR "Hypogonadism/mortality" (Majr) OR "Hypogonadism/therapy" (Majr)) OR ("Hypogonadism/classification"(Mesh:NoExp) OR "Hypogonadism/drug therapy" (Mesh:NoExp) OR "Hypogonadism/mortality" (Mesh:NoExp) OR "Hypogonadism/therapy" (Mesh:NoExp)). The search was filtered to include only studies published after 2010.

Eligibility Criteria and study selection

Two investigators (JL, PS) screened each article’s title and abstract to determine eligibility independently first. Then the studies included by both reviewers were compared and disagreements were resolved by consensus. When the consensus could not be reached between the two investigators, the third independent investigator (UD), who did not participate in the original screening, decided the eligibility. The following inclusion criteria were utilized to screen the results: 1) Full-text articles available, 2) Studies published in the English language, 3) Observational and randomized controlled trials (RCT) that explore the relationship between TRT and MI, 4) Studies conducted on human males who have lower testosterone levels irrespective of age, ethnicity, or study location, 5) Included studies even if MI was not the principal endpoint, and 6) Studies including hypogonadal men with <300ng/dL testosterone at baseline.

The exclusion criteria included were: 1) Editorials, posters, and animal studies, 2) Irrelevant studies, 3) Studies conducted only including specific populations with certain comorbidities e.g diabetics, post-MI, heart failure, or any other, 4) Studies that did not observe MI incidence was excluded, and 5) Studies using androgens other than testosterone. Only studies meeting the above criteria were evaluated for eligibility in the final review.

Data Extraction

Two independent investigators (JL, PS) performed data extraction from the selected studies. All of the following variables were investigated using a standardized recording tool: study design, number of study participants, baseline characteristics of participants including comorbidities and testosterone level, mode of treatment and treatment dose, the mean follow-up in each group of participants, study outcomes, and whether the study was funded by a pharmaceutical company.

Quality Assessment Tools

Two investigators evaluated the risk of bias, using the Newcastle-Ottawa questionnaire for the observational studies (Table 1) and Cochrane risk-of-bias tool (Table 2) for clinical trials. We only included studies that had scores six and above in the Newcastle-Ottawa questionnaire for the observational studies and RCT, we only included studies that were judged as “low-risk” of bias in each of the domains. Disagreement was resolved by consensus.

**Table 1:** Quality assessment of observational studies using the Newcastle-Ottawa questionnaire

Study	Selection	Comparability	Outcome	Overall (max 9)
Traish et al. [18]	★★★	★	★★	6, Good
Vigen et al. [19]	★★★	★	★★	6, Good
Cheetham et al. [20]	★★★★	★	★★	7, Good
Ramasamy et al. [21]	★★★	★	★★	6, Good
Maggi et al. [22]	★★★★	★	★★★	8, Good
Pantalone et al. [23]	★★★★	★	★★	7, Good

**Table 2:** Quality assessment of RCT using the Cochrane risk-of-bias tool

RCT	Selection bias	Reporting bias	Performance bias	Detection bias	Attrition bias
Basaria et al. [24]	Low Risk	Low Risk	Low Risk	Low Risk	Low Risk
Basaria et al. [25]	Low Risk	Low Risk	Low Risk	Low Risk	Low Risk

Data analysis

We performed the final statistical analysis using the Review Manager (RevMan) version 5.4 (The Nordic Cochrane Centre, The Cochrane Collaboration, Copenhagen). For dichotomous results, we calculated the risk and OR and 95% CI using the random-effects model and the Mantel-Haenszel method. Statistical significance was described when the two-sided p-value was <0.05.

The heterogeneity was calculated using I^2^ statistics. As explained in the *Cochrane Handbook for Systematic Reviews* [26], I^2^ >50% was considered to be substantial heterogeneity. We carried out sensitivity analysis for significant heterogeneity.

Results

Literature search and study selection

**Figure 1:**
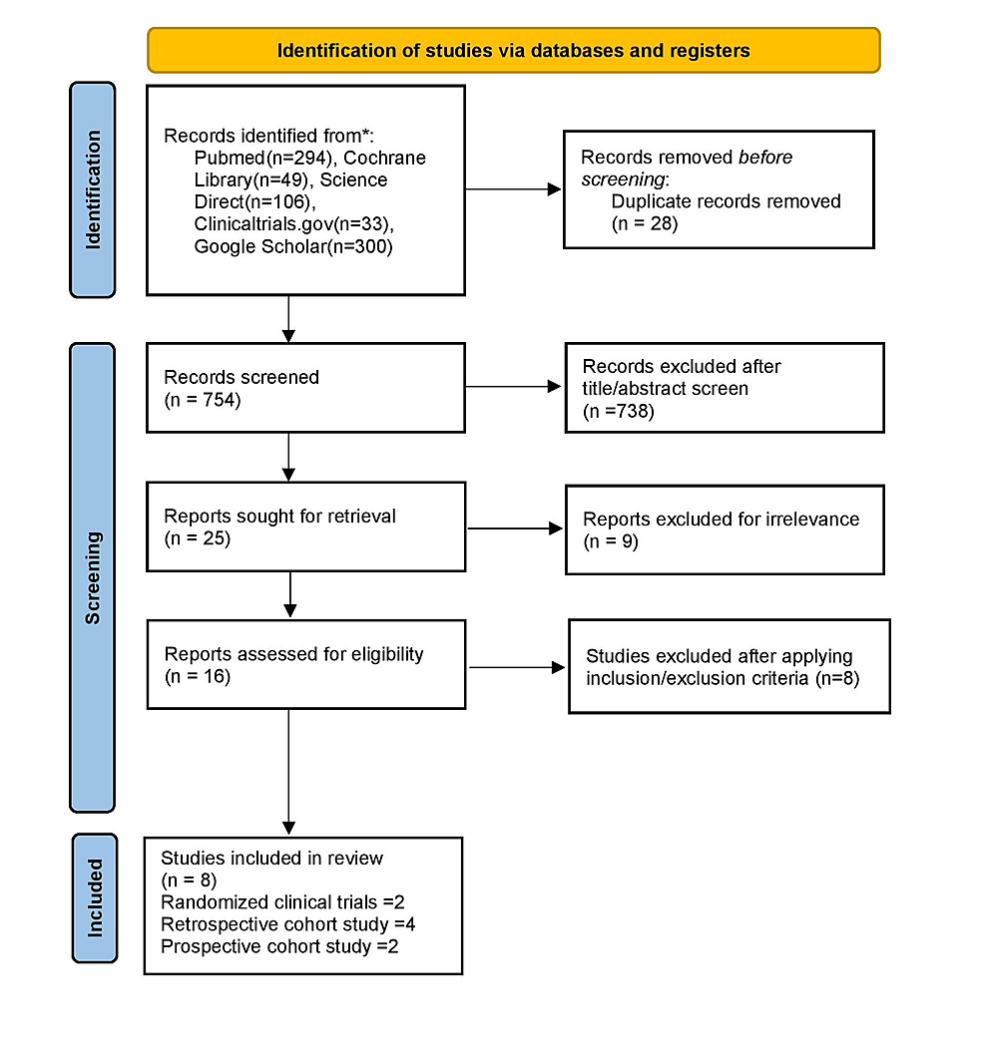
PRISMA flow diagram

Our search strategy yielded the following: Pubmed using MeSH keys words filtering to include studies from 2010-2021, full text available, article type to include clinical trial, and RCT yielded 294, ScienceDirect yielded 106 after filtering research articles only and studies after 2010, Clinicaltrials.gov yielded 33, Cochrane library of clinical trials yielded 49, Google Scholar 300 of the first sorted by relevance was added out of the total 5960. The search strategy was used totaling 782 in all. After applying inclusion and exclusion criteria, and duplication removal, 16 full-text articles were found to be potentially eligible. Of these, eight articles were identified as suitable for our research question. This is represented in Figure 1.

Baseline Characteristics of Included Studies

The characteristics of the included studies are shown in Table 3. Out of the eight studies included in our study, two studies were RCT, two were prospective cohort and the remaining four out of the eight studies were retrospective cohort studies.

**Table 3:** Baseline characteristics of included studies T = testosterone, DM = diabetes mellitus, MI = myocardial infarction, CAD = coronary artery disease, RCT = randomized controlled trial, N/A = not available, CHF = congestive heart failure, OSA = obstructive sleep apnea, COPD = chronic obstructive pulmonary disease, CVD = cardiovascular disease, CV: cardiovascular

Study	Study Design	Patient mean age: TRT group	Patient mean age: Control	Total sample size (n)	Number of patients taking TRT (n)	Number of patients in the control group (n)	Mean baseline total testosterone levels (Treatment)	Mean baseline total testosterone levels (Control)	Testosterone dose/mode	Co-morbidities in the TRT group		Co-morbidities (%) : control		Mean follow-up period	Number of MI in treatment group	Number of MI in Control Group	Conclusion regarding CV events and TRT
Traish et al. [18]	Observational, Prospective, Cohort Study	57.4	64.8	656	360	296	9.8mmol/L	9.6mmol/L	Parenteral T undecanoate 1000mg/12 weeks	DM1	6.7%	DM1	1.4%	7 years	0	31	Lower Risk of CV events in TRT group
										DM2	31.4%	DM2	39.5%				
										Previous MI	11.1%	Previous MI	7.8%				
										Previous Stroke	1.7%	Previous Stroke	8.1%				
										Prior CAD:	11.1%	Prior CAD	22.6%				
Basaria et al. [24]	RCT	66.9	68.3	306	155	151	8.86mmol/L	8.87mmol/L	75mg of Testosterone gel – prematurely stopped at 6months due to increased adverse effects	Obesity	25.8%	Obesity	28%	6 month treatment followed by 3month observation period	3	2	Increases risk of CV events in TRT group
										DM	14.2%	DM	16%				
										Hypertension	45.8%	Hypertension	38%				
										Hyperlipidemia	51.6%	Hyperlipidemia	77%				
										Prior CAD	15.5%	Prior CAD	14.7%				
Vigen et al. [19]	Retrospective Cohort	63.8	60.6	8704	1223	7486	5.06mmol/L	5.96mmol/L	Gel/patch/Injection Dose N/A	DM	53.2%	DM	53.2%	27.5 months	23	420	Increases risk of CV events in TRT group
										Obesity	53.9%	Obesity	57.5%				
										Hypertension	92.9%	Hypertension	90.0%				
										Hyperlipidemia:	88.3%	Hyperlipidemia	85.9%				
										Cerebrovascular Disease	16.3%	Cerebrovascular Disease	11.1%				
										Peripheral vascular disease	19.5%	Peripheral vascular disease	16.4%				
										COPD	21.7%	COPD	18.6%				
										CHF	24.4%	CHF	18.2%				
										OSA	26.4%	OSA	27.9%				
										Previous MI	24.3%	Previous MI	20.3%				
Cheetham et al. [20]	Retrospective Cohort	58.4	59.8	44335	8808	35527	all patients <8.65mmol/L	all pateints <8.65mmol/L	N/A	Hypertension:	44.5%	Hypertension:	44.2%	4.2 median years in the TRT treatment group and 3.2 median years in the control group	204	962	Decreases risk of CV events in TRT group
										CHF:	1.3%	CHF:	2.0%				
										Dyslipidemia:	50.3%	Dyslipidemia:	50.9%				
										COPD:	4.3%	COPD:	4.5%				
										OSA:	2.3%	OSA:	2.3%				
										Diabetes:	23%	Diabetes:	23%				
										Obesity:	30.9%	Obesity:	32%				
Ramasamy et al. [21]	Retrospective	74	73	217	153	64	all patients <300ng/dL	all patients<300ng/dL	Injection = 53 Gel = 47 Pellets = 53	Charlson Cormorbidity Index = 5.1		Charlson Cormorbidity Index = 5.3		3.8 median years in the TRT treatment group and 3.4 median years in the control group	1	0	No difference in CV events between the control and TRT group
Maggi et al. [22]	Prospective Cohort	58.9	59.7	999	750	249	8.3nmol/L	9.4nmol/L	Gel = 68% Injections = 31% Oral = 2%	DM	28.4%	DM	29.7%	At least 2 years	11	2	No difference in mortality or the risk of CV events
										CHF	1.3%	CHF	2.4%				
										Hypertension	47%	Hypertension	45%				
										Cerebrovascular disease	1.9%	Cerebrovascular disease	3.6%				
										Previous MI:	4.4%	Previous MI:	6.4%				
										Peripheral vascular disease	3.9%	Peripheral vascular disease	4.8%				
Basaria et al. [25]	RCT	74	74	209	106	103	250ng/dL	236ng/dL	Testosterone Gel 100mg x 1 for 6months first 2 weeks after dose adjusted +/- 5g if <17.4nmol/L vs 34.7nmol/L	Hypertension:	85%	Hypertension:	78%	6months	3	0	No conclusion
										DM:	24%	DM:	27%				
										Hyperlipidemia:	63%	Hyperlipidemia:	50%				
										Obesity:	45%	Obesity:	49%				
										CVD:	53%	CVD:	48%				
Pantalone et al. [23]	Retrospective Cohort	55 (median years)	55 (median years)	375	165	210	179ng/dL (median)	173ng/dL(median)	N/A	Hypertension:	67.3%	Hypertension:	67.6%	3.4 median years in the TRT treatment group and 2.7 median years in the control group	4	3	No difference in CV events between the control and TRT group
										DM:	40.6%	DM:	41.9%				
										CVD	20.0%	CVD	17.1%				

Outcomes

**Figure 2:**
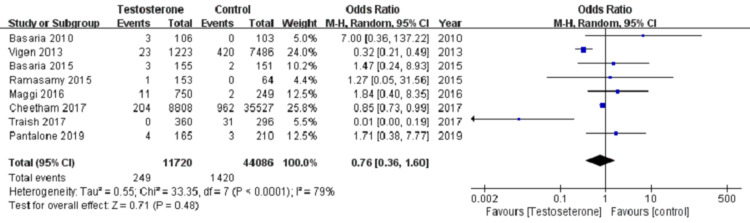
Forest plot comparing odds ratio of myocardial infarction for testosterone therapy vs control The Cochran-Mantel-Haenszel method and the random-effects model were used to calculate the pooled odds ratio.

**Figure 3:**
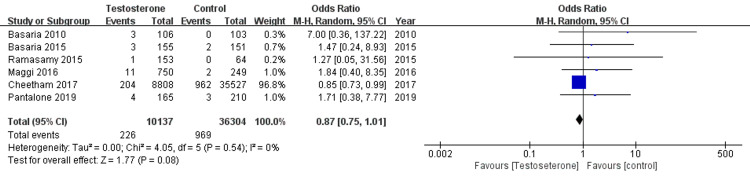
Forest plot comparing odds ratio myocardial rates for testosterone replacement therapy vs. control After removing two studies by performing sensitivity analysis, the pooled data show low heterogeneity. I^2^= 0%

**Figure 4:**
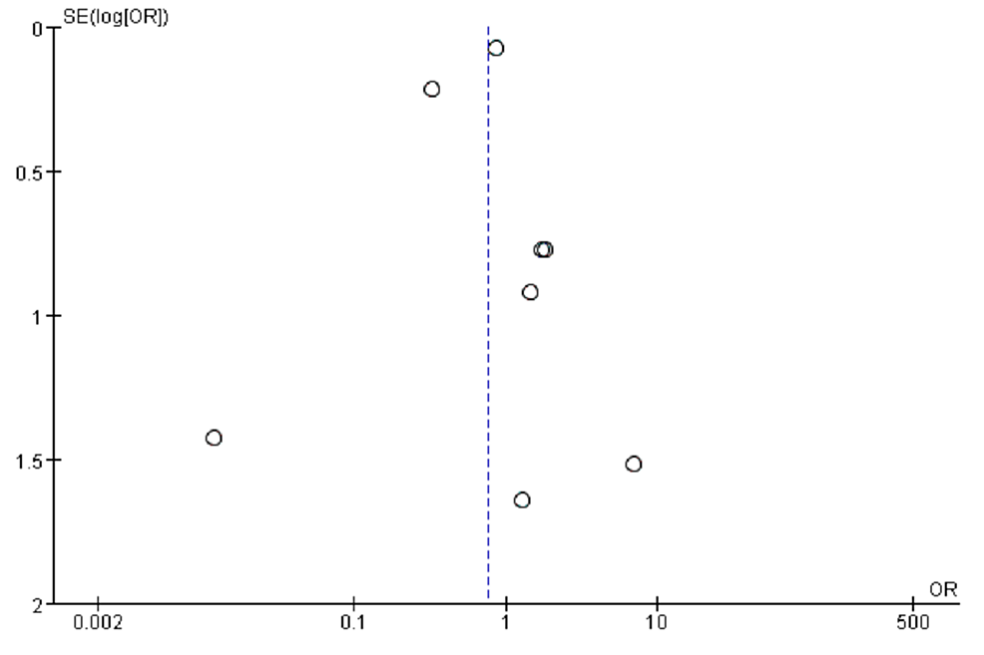
Testosterone replacement therapy and myocardial infarction risk SE: standard error, OR: odds ratio

The pooled data from eight studies showed MI incidence in 249 out of 11,720 (2.1%) in the TRT group and 1420 out of 33,086 (4.3%) in the control group. Figure 2 reveals pooled estimate which showed no statistically significant association of TRT and MI compared to the control group. (OR = 0.76, 95% CI 0.36-1.31; p=0.48). The model revealed high heterogeneity with I^2^ =79%. With sensitivity analysis, by excluding Vigen et al. and Traish et al., the pooled data was able to achieve low heterogeneity with I2 = 0%. The newly pooled data from six studies showed MI incidence in 226 out of 10,137 (2.2%) in the TRT group and 969 out of 36,304 (2.7%) in the control group. Figure 3 reveals no statistical significance in the association between TRT treatment and MI compared to the control group. (OR =0.87, 95% CI 0.75-1.01; P =0.08). The funnel plot was used to assess the publication bias. A visual assessment of Figure 4 showed slight asymmetry, suggestive of possible publication bias, thus we used a random effect model to analyze the selected studies.

Discussion

A meta-analysis by Maggi, et. al. [27], which included 70 studies, has indicated that patients with cardiovascular disease have significantly lower testosterone levels and higher 17-beta estradiol levels. The higher incidence of overall mortality and cardiovascular-related mortality in patients were found in lower baseline testosterone levels in longitudinal studies. Also, TRT was linked to a substantial increase in treadmill test length as well as the time to 1mm ST-segment depression. Other results from retrospective epidemiological studies support this conclusion given by the meta-analysis [28,29].

However, simply replacing testosterone with TRT in patients with a low blood level of testosterone does not necessarily guarantee that the risk of cardiovascular disease and/or overall mortality will be lowered. It is unclear whether low testosterone and cardiovascular risk have a true cause-and-effect relationship. On the other hand, low testosterone may be just an indicator of overall health. The Testosterone in Older Men (TOM) trial [25] was stopped at six months due to increased adverse events, more specifically cardiovascular-related events, in the treatment with the testosterone gel group. However, there are some controversies regarding the results of this study. The TOM trial had a small number of participants (209), and its study population consisting of only older men with limitations in mobility and a high prevalence of comorbidities. Also, the study was not designed to study cardiovascular events as a primary outcome. However, with these growing concerns, in 2015, FDA drug safety communication issued a warning against using testosterone treatments to treat low testosterone due to aging. It had mandated a labeling change to inform of an elevated risk of MI and stroke with its use [30]. In our meta-analysis study, no statistically significant difference was found in the overall incidence of MI in hypogonadal men receiving testosterone therapy compared to hypogonadal men not taking any testosterone therapy. This finding is coherent with the 2014 statement from the European Medicine Agency (EMA), which stated that there is no clear evidence that testosterone medications raise cardiovascular risk in hypogonadal men [31]. The link between testosterone therapy and heart disease is complicated. Testosterone medication can raise or decrease the risk of adverse cardiovascular events through a variety of physiological routes. Testosterone therapy can cause edema, hypertension, and heart failure by increasing salt and water retention [32,33]. Testosterone can upregulate the expression of thromboxane A2 receptors, resulting in platelet aggregation and an increase in thrombotic events such as MI [34]. Left ventricular hypertrophy, systolic and diastolic dysfunction from testosterone are also possible [35].

In contrast, there are other pathways in which testosterone may potentially reduce the risk of cardiovascular disease. Testosterone therapy may improve cardiovascular health by lowering fat mass, lipid profile, and insulin sensitivity. Furthermore, testosterone may have anti-inflammatory properties, which had been shown to reduce the thickness of carotid intima-media [36]. As there are various ways in which testosterone can influence the cardiovascular system, conducting more extensive studies on the biological mechanism of testosterone is imperative.

Duration of Testosterone Therapy and Cardiovascular Risk

In the intention to treat an observational cohort study by Wallis et al. [37], which included 40,289 men over the age of 66, concluded that long-term TRT (median 35 months) was linked to lower mortality and cardiovascular events. In contrast, short-term TRT (median two months) increased the risk of mortality and cardiovascular events. However, this study lacked data on the baseline testosterone level in their studied subjects as well as the reasoning behind the testosterone therapy on their patients.

Traish et al. [18] also concluded with similar results. This study followed 360 hypogonadal men treated with testosterone for eight years and compared against 296 men with hypogonadism without treatment for the same follow-up period. This study also showed that mortality related to cardiovascular disease was significantly lower in the testosterone group. Moreover, the anthropometric parameters and other cardiovascular risk factors such as blood glucose levels were also compared in this study. There was a reduction in waist circumference and BMI in the testosterone treatment group compared to the untreated group, as well as a reduction in blood glucose shown through measurement of HbA1c levels in the testosterone treatment group. However, as this study included men with Klinefelter syndrome and other primary hypogonadism patients who had to receive testosterone therapy, its treatment group was considerably younger than the control group and potential selection bias.

Normalization of Testosterone Levels and the Risk of Myocardial infarction

Sharma et al. [38] made an outcome distinction in the TRT group, differentiating the outcome on whether the patient was normalized to normal testosterone level or had failed to achieve normal testosterone level with TRT. In this retrospective study, patients were divided into these three groups: no treatment, TRT with normalization of serum testosterone, TRT without normalization of serum testosterone. In comparison between the TRT treatment group who had failed to achieve normal testosterone level and the no treatment group, there was no statistical difference in MI, all-cause mortality, and stroke. On the other hand, patients with normalization of testosterone after TRT had a lower risk of MI, all-cause mortality, and stroke, compared to no treatment group or TRT without normalization of testosterone group. In comparison with normalized treated with untreated men for MI risk, the adjusted hazard ratio was 0.76 with a CI of 0.63-0.95. Comparing MI risk for normalized TRT group compared with those who did not have normalized testosterone levels after TRT, the adjusted hazard ratio was 0.82 with CI 0.71-0l.95.

These findings suggest that normalization of testosterone with TRT may be the key factor in the association between TRT and the risk of MI. As this study only includes men without prior MI and stroke, more studies on other populations are needed to establish a more clear association with the normalization of testosterone with TRT in hypogonadal men and the lowered risk of MI and overall mortality.

Limitations

There are certain limitations to this study, which are mostly attributed to the limitations of the studies that were included. First, we only included only eight studies that met our inclusion criteria. The result produced with all eight studies produced high heterogeneity. However, with sensitivity analysis and including only six out of the eight studies, the heterogeneity of the analyses was found to be low (0%). We have included retrospective observational studies and non-randomized assignments in these studies that may have offered certain bias risks. For example, testosterone therapy itself was not free for most patients, there could be some selection bias present in selected studies as some patients may not have decided to receive testosterone therapy due to its cost. However, we do not believe that this would have a significant impact on our findings. Our meta-analysis was based on pooled data. We did not have access to certain crucial information about the individual participants who suffered MI. The eight studies selected were not designed or powered to address the risk of MI with testosterone therapy in hypogonadal men specifically. To our knowledge there are no studies that investigated this topic specifically, meeting our study selection criteria to this date. However, most of the selected studies were investigating cardiovascular events as a primary outcome, and we believe that our study results should be considered hypothesis-generating. We do not believe that our results are definitive and that it provides sufficient evidence for the safety of testosterone usage in hypogonadal men with the risk of MI. However, the findings from this study may help doctors discussing potential benefits and risks in initiating TRT in hypogonadal men.

## Conclusions

In conclusion, this meta-analysis found no statistically significant difference between the risk of MI and testosterone therapy in hypogonadal men compared to non-treated hypogonadal men. As there are no long-term prospective placebo-controlled trials to investigate the MI risks and benefits of testosterone therapy in men with hypogonadism, we do not believe that this finding provides definitive evidence for cardiovascular safety in the use of testosterone replacement TRT in hypogonadal men. However, findings from this paper may aid doctors counseling hypogonadal men about the benefits and risks of TRT before initiating the treatment. Moreover, our study warrants the need for more future RCTs measuring more specific parameters such as the duration of testosterone therapy, the methods of testosterone therapy, and the normalization of testosterone level in hypogonadal men with greater population size, which could produce more solid results, allowing more definitive conclusions to be made on this topic.
